# Metronomic Chemotherapy in Dogs and Cats: Mechanisms, Indications, and Clinical Perspectives

**DOI:** 10.3390/cancers17203318

**Published:** 2025-10-14

**Authors:** Rafael Costa Bitencourt, Giovanna Gabrielle Cruvinel, Verônica Maria Teixeira de Castro Terrabuio, Laís Calazans Menescal Linhares, Guilherme Andraus Bispo, Letícia Abrahão Anai, Márcia Ferreira da Rosa Sobreira, Annelise Carla Camplesi, Andrigo Barboza De Nardi, Aureo Evangelista Santana

**Affiliations:** 1 Department of Veterinary Clinic and Surgery, School of Agricultural and Veterinary Sciences, São Paulo State University, Via de Acesso Professor Paulo Donato Castellane S/N–Vila Industrial, Jaboticabal 14884-900, SP, Brazil; giovanna.g.cruvinel@unesp.br (G.G.C.); terrabuio.c.veronica@gmail.com (V.M.T.d.C.T.); lais.menescal@unesp.br (L.C.M.L.); leticia.anai@unesp.br (L.A.A.); marcia.fr.sobreira@unesp.br (M.F.d.R.S.); annelise.camplesi@unesp.br (A.C.C.); andrigo.barboza@unesp.br (A.B.D.N.);; 2 Department of Clinic, Surgery, and Animal Reproduction, School of Veterinary Medicine, São Paulo State University, Clóvis Pestana Street, 793–Dona Amélia, Araçatuba 16050-680, SP, Brazil; g.bispo@unesp.br

**Keywords:** malignancies, treatment, veterinary oncology

## Abstract

Metronomic chemotherapy (MC) represents an emerging strategy in veterinary oncology which involves the continuous or regularly scheduled administration of low-dose chemotherapeutic agents. Unlike conventional protocols known as maximum tolerated dose chemotherapy (MTDC), MC aims to inhibit tumor angiogenesis, stimulate antitumor immune responses, and delay or prevent the emergence of drug resistance. This review is structured into three key sections: the mechanisms of action of MC; its clinical indications in dogs and cats, particularly for advanced or treatment-resistant cancers; and reported outcomes regarding efficacy, safety, and tolerability. Additionally, we explore the growing interest in combining MC with other therapies, as well as the challenges and future directions for optimizing its use. Current evidence suggests that MC is a promising and well-tolerated option for managing various malignancies—such as carcinomas, sarcomas, and hemangiosarcomas—especially in patients who are not candidates for MTDC.

## Simple Summary

Cancer is a common and serious disease in dogs and cats. Conventional chemotherapy, while often effective, can cause significant side effects that reduce the quality of life for pets. Therefore, metronomic chemotherapy (MC) is an emerging approach that uses continuous low-dose treatment to slow tumor growth while minimizing adverse effects. This review explains how MC works, summarizes its use in veterinary patients, and highlights outcomes reported across different types of cancer. By compiling recent research, we aim to demonstrate how MC can serve as a safer, more affordable, and practical option for cancer treatment in animals, helping to guide future studies and improve veterinary oncology care.

## 1. Introduction

**Figure 1:**
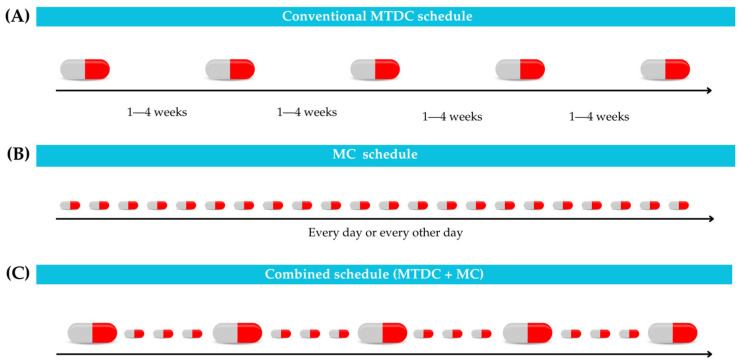
Different chemotherapy schedules. (**A**) In maximum tolerated dose chemotherapy (MTDC), the drug is administered at longer intervals via various routes, including oral, intravenous, or subcutaneous. (**B**) Metronomic chemotherapy (MC) facilitates continuous administration of the drug in low doses. (**C**) Combined schedule integrates MTDC + MC.

Metronomic chemotherapy (MC) is characterized by the continuous administration of low-dose chemotherapeutic agents, such as cyclophosphamide, lomustine, and thalidomide, with activities aimed at inhibiting tumor angiogenesis and modulating the immune system response [1,2,3,4,5]. It has surfaced as a viable alternative to the conventional maximum tolerated dose chemotherapy (MTDC), aiming to control tumors while minimizing the adverse effects of chemotherapeutic agents. The MTDC depends on administering the highest dose tolerable to dogs and cats, which may lead to significant toxicities such as gastrointestinal and hematological complications, potentially requiring hospitalization [1,2,3]. Consequently, the therapeutic window for the MTDC should be extended, and it should be administered at longer intervals to reduce its toxicity, as illustrated in Figure 1A [6].

Considering these limitations, MC becomes a notably valuable option for patients who are unsuitable for the MTDC, such as those with renal or hepatic dysfunction, or in cases where the efficacy of conventional therapy has plateaued [1]. In addition, the financial burden of the high-cost MTDC regimens may be challenging for many pet owners, rendering the more affordable and potentially safer approach of MC a preferable alternative [4,5]. Therefore, MC permits a much narrower therapeutic window than MTDC and allows for reduced tumor exposure during chemotherapy-free periods, as shown in Figure 1B. Moreover, combining MC protocols with MTDC can enhance the efficacy of anti-tumor therapy in selected cases, as illustrated in Figure 1C [6].

Metronomic chemotherapy is particularly effective for advanced or inoperable tumors, as well as for patients with contraindications to MTDC. It is also used as an adjuvant therapy to prevent tumor recurrence. The protocols are easy to administer, often relying on oral medications, and they can be combined with other therapies for improved outcomes [1,7]. Since its introduction in 2007, MC has been recognized as a relatively novel concept in the field. Although it holds great promise, further rigorous scientific investigation is necessary to fully validate its efficacy and expand its clinical indication in veterinary oncology [1,8,9].

Given the above, this review aimed to provide a comprehensive analysis of MC in dogs and cats [10] and is organized into three core sections: the mechanisms of action of MC; its clinical indications in dogs and cats, including tumor-specific indications and patient selection; and an assessment of therapeutic outcomes, encompassing efficacy, tolerability, and safety. Additionally, it addresses the potential for combination therapies, current challenges, and future directions to enhance the efficacy and safety of MC in veterinary oncology.

## 2. Mechanisms of Action

Currently, MC is recognized as a multifaceted therapeutic approach. Its primary mechanisms of action are categorized into four main groups: (1) inhibition of neovascularization, (2) modulation of the immune response, (3) direct effects on tumor cells and cancer stem cells (CSCs), and (4) promotion of tumor dormancy [1,4,7,8,10].

### 2.1. Inhibition of Neovascularization

Metronomic chemotherapy exerts antiangiogenic effects by targeting activated endothelial cells within tumor vasculature. These cells are highly sensitive to sustained low-dose chemotherapy, leading to reduced proliferation and impaired blood vessel formation. This is accompanied by the downregulation of pro-angiogenic factors (e.g., VEGF) and upregulation of antiangiogenic mediators (e.g., thrombospondin-1), ultimately reducing tumor perfusion and metastatic potential [1,8].

### 2.2. Modulation of the Immune Response

A key immunological effect of MC is the selective depletion of regulatory T cells, which promotes the enhanced activity of effector T cells and natural killer cells. MC also facilitates dendritic cell maturation and antigen presentation, contributing to a more effective antitumor immune response [1,10].

### 2.3. Effects on Tumor Cells and Cancer Stem Cells (CSCs)

At subtoxic concentrations, MC induces apoptosis and cell cycle arrest in tumor cells. It also disrupts the self-renewal and proliferation of CSCs, reducing the risk of recurrence and chemoresistance [8].

### 2.4. Promotion of Tumor Dormancy

Through sustained cytostatic pressure, MC can induce a state of tumor dormancy characterized by suppressed angiogenesis, limited proliferation, and immune containment. This may delay progression and prolong survival, particularly in chronic or palliative treatment settings [8,10].

## 3. Pharmacokinetics and Pharmacodynamics of Commonly Used Metronomic Chemotherapy Agents

Metronomic chemotherapy requires careful selection of agents based on their pharmacokinetic and pharmacodynamic properties to ensure sustained therapeutic activity with reduced toxicity [7]. Unlike conventional chemotherapy, which often depends on maximum tolerated doses to induce tumor cell death, MC relies on alternative mechanisms such as antiangiogenesis and immune modulation [1].

Therefore, drugs that require high peak plasma concentrations to achieve efficacy—such as doxorubicin, vincristine, and carboplatin—are not suitable for MC due to their pharmacologic profiles and potential for cumulative toxicity. These agents are reserved for traditional MTDC protocols, while MC favors compounds with favorable oral bioavailability, longer half-lives, and the ability to exert cytostatic or modulatory effects at low concentrations [11].

### 3.1. Cyclophosphamide

Cyclophosphamide is an alkylating agent activated in the liver into phosphoramide mustard and acrolein, which exerts cytotoxic effects by cross-linking DNA and inhibiting cell replication [11].

It is well absorbed orally, with wide tissue distribution except to the central nervous system (CNS) and has a half-life of 4–12 h after IV use. The drug and its metabolites are eliminated mainly by the kidneys over 48–72 h [7,11].

Acrolein is responsible for bladder toxicity, making hydration and morning dosing essential to reduce the risk of sterile hemorrhagic cystitis [11].

Adverse effects include dose-dependent myelosuppression (leukocyte nadir at ~7 days), sterile hemorrhagic cystitis—associated with acrolein—and, less commonly, gastrointestinal signs and alopecia. Preventive strategies (e.g., morning dosing, hydration, frequent voiding, and IV furosemide) reduce cystitis risk to <1%. Management involves discontinuation of cyclophosphamide and diuretics.

### 3.2. Chlorambucil

Chlorambucil is a slow-acting alkylating agent that exerts its cytotoxic effect by cross-linking DNA strands, impairing replication and leading to apoptosis in rapidly dividing cells [11].

After oral administration, it is rapidly absorbed, reaching peak plasma levels within 1 h. Approximately 99% is bound to plasma proteins. It undergoes hepatic metabolism to form the active metabolite phenylacetic acid mustard [11].

The elimination half-life is 1.5 h for chlorambucil and 2.4 h for its metabolite. Renal excretion accounts for up to 60% of the dose within 24 h [11].

It is generally well tolerated, with cumulative myelosuppression being the most common adverse effect in cats and dogs. Chlorambucil may be used as an alternative to cyclophosphamide in cases of hemorrhagic cystitis.

### 3.3. Thalidomide

Thalidomide is an oral immunomodulatory and antiangiogenic agent that reduces tumor vascularization and inhibits pro-inflammatory cytokines such as TNF-α and IL-6 [12,13].

It is absorbed variably depending on feeding status, with higher exposure and delayed absorption when given with food. The drug reaches peak plasma concentrations within 3 to 10 h and has a half-life ranging from 4.8 to 17 h [13].

Thalidomide is well tolerated at doses of 5–10 mg/kg/day, achieving plasma levels consistent with therapeutic ranges observed in human oncology [13].

Despite its historical teratogenicity, adverse effects in dogs are infrequent at therapeutic doses; however, caution is advised due to the unknown excretion route in this species, requiring careful handling of excreta [12,13].

### 3.4. Piroxicam

Piroxicam is a nonsteroidal anti-inflammatory drug (NSAID) with antitumor activity mainly related to COX-2 inhibition, which reduces inflammation and angiogenesis in tumor tissues [11].

The drug is well absorbed after oral administration, with peak levels in about 3 h and a half-life of approximately 12 h. It is highly protein-bound, metabolized in the liver, and excreted via the urine [11].

Adverse effects include gastrointestinal toxicity and nephrotoxicity, especially in cats, requiring periodic monitoring of both renal function and hematocrit [11].

### 3.5. Doxycycline

Doxycycline is a lipophilic tetracycline with high oral bioavailability and wide tissue distribution, including tumors [14].

In veterinary oncology, its pharmacodynamics include time-dependent inhibition of protein synthesis and additional anti-inflammatory, immunomodulatory, and anti-angiogenic effects [14].

These properties support its potential use in MC as an adjunct to conventional cancer treatment [14].

In dogs and cats, adverse effects associated with prolonged administration include gastrointestinal signs—more frequently observed in cats—and, in young animals, permanent staining of unerupted teeth.

Table 1 provides a summary of the pharmacological characteristics of commonly used MC agents in veterinary oncology, including route of administration, dosing, half-life, and key toxicities.

## 4. Clinical Indications of Metronomic Chemotherapy

Historically, the clinical indication of MC began as a rescue therapy for human patients who were refractors to MTDC protocols. Even in this unfavorable scenario, MC controlled the neoplasm and extended disease-free periods [15,16]. Consequently, research into the use of MC in different protocols has expanded, including combinations with various local and systemic therapies.

**Scheme 1:**
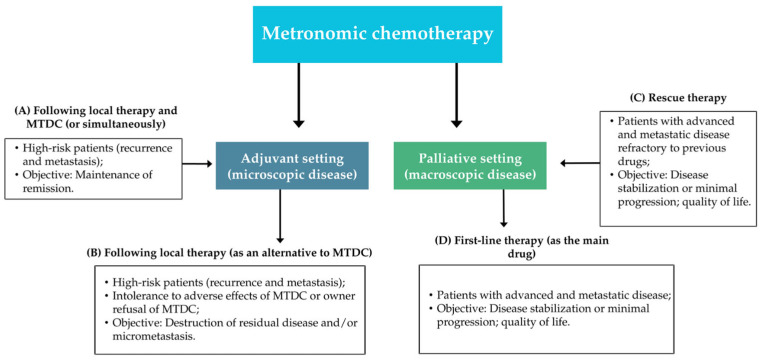
Various clinical scenarios in which the use of metronomic chemotherapy (MC) has been evaluated along with the respective objectives of its indication. Chemotherapy treatment can be combined with other drugs (e.g., NSAIDs, thalidomide, etoposide, and targeted therapy). (**A**) Following local therapy and maximum tolerated dose chemotherapy (MTDC) (or simultaneously) [18,19,20,21,22]; (**B**) following local therapy (as an alternative to MTDC) [2,17,23,24,25,26,27,28,29]; (**C**) rescue therapy [30,31]; and (**D**) first-line therapy (as the main drug) [2,32,33,34,35,36].

The first clinical study to evaluate MC in veterinary medicine was conducted on dogs with stage II splenic hemangiosarcoma, treated with a MC protocol comprising cyclophosphamide, etoposide, and piroxicam [17]. In this study, MC was instituted not as a palliative therapy but as an adjuvant to surgery, proposing it as a potential alternative to conventional chemotherapy. Since then, numerous clinical studies have explored the therapeutic potential of MC in various settings. Its use can be classified into two main modalities: palliative therapy, targeting macroscopic disease, and adjuvant therapy, aimed at microscopic disease. This classification is further detailed in Scheme 1, which summarizes the clinical scenarios and treatment goals associated with each modality.

In veterinary studies, the therapeutic response to MC has been assessed using standard definitions. Complete remission (CR) is defined as the total disappearance of all detectable tumor lesions sustained for a minimum period. Partial remission (PR) refers to a ≥30% reduction in the sum of the longest diameters of measurable lesions, with no new lesions or progression of existing ones. Stable disease (SD) denotes the absence of both sufficient reductions to meet PR and significant progression. Progressive disease (PD), on the other hand, is characterized by a ≥20% increase in total tumor burden or the emergence of new lesions [30].

Considering that cancer is a chronic disease, MC for macroscopic disease primarily aims at tumor control and clinical benefit and is thus considered a palliative therapy. Although it may result in a reduction in tumor size, objective response rates (CR and PR) are low, varying by 3–16%. However, approximately 30–76% of these patients achieve disease stabilization (or minimal progression), with a clinical benefit (CR, PR, and SD) of up to 92% [2,30,31,33].

The utilization of MC in treating macroscopic disease can be indicated as a salvage therapy or as a first-line therapy in selected cases. As a salvage therapy, it is recommended for cases that have not responded to conventional first-line therapies, such as inoperable bladder carcinomas refractory to MTDC [37]. When used as a first-line therapy, MC is primarily documented in the treatment of metastatic and/or advanced-stage tumors of various histological types, including primary lung carcinoma, inflammatory breast carcinoma, osteosarcoma, hepatocellular carcinoma, and oral melanoma [2,32,33,34,35,36]. In these instances, patient selection depends largely on the impracticality of local treatment due to the advanced stage of disease (e.g., unresectable or metastatic tumors) and/or patient or guardian limitations (e.g., comorbidities or the guardian’s refusal of standard therapy).

**Scheme 2:**
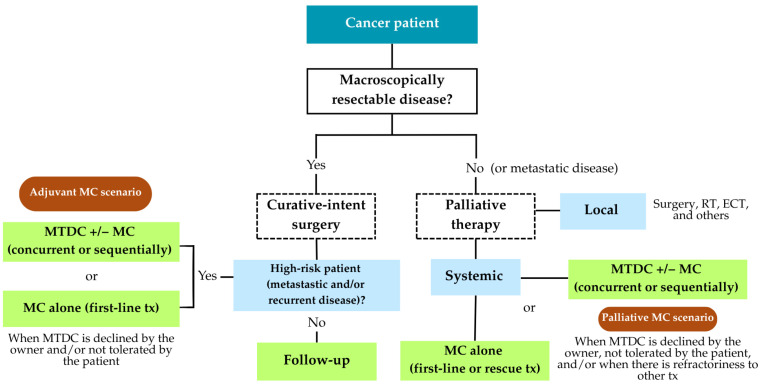
Decision-making algorithm for the clinical use of metronomic chemotherapy (MC)—Palliative vs. Adjuvant. RT: radiotherapy; ECT: electrochemotherapy; MTDC: maximum tolerated dose chemotherapy; MC: metronomic chemotherapy; tx: treatment.

A summary of the decision-making process for the clinical use of MC—distinguishing palliative and adjuvant indications—is presented in Scheme 2.

Meanwhile, MC as an adjunct to first-line therapies primarily targets microscopic disease in patients with tumors possessing high metastatic potential and recurrences, aiming to eliminate possible residual tumor foci and micrometastases. In this context, metastatic neoplasms (e.g., visceral hemangiosarcoma, stage IV and V breast carcinomas, and osteosarcoma) and those that are challenging to control locally (e.g., soft tissue sarcomas) are prominent. MC can be applied concurrently with or subsequent MTDC, generally following the local therapy of choice (surgery, radiotherapy [RT], and/or electrochemotherapy), or it can be an alternative to MTDC after local therapy is established [20,21,22,23,24,26,27].

Importantly, while cyclophosphamide remains one of the most frequently used agents in MC protocols, its urothelial carcinogenic potential raises significant clinical concern—particularly in patients with, or predisposed to, urothelial carcinoma (UC). As highlighted in human oncology, cyclophosphamide has been linked to bladder tumor development and recurrence [38]. Although data in veterinary patients are limited, this risk should not be overlooked. Thus, in clinical scenarios involving UC, the use of cyclophosphamide represents a therapeutic paradox—where the agent intended to treat the neoplasm may simultaneously potentiate its progression. Given this contradiction, chlorambucil and other safer alternatives should be prioritized in MC protocols for such cases, and the choice of agent should be guided by both oncologic evidence and patient safety considerations [39,40].

Thus, the decision to use MC, in its various forms, must consider several disease- and patient-specific factors: (1) tumors with high metastatic potential and/or recurrence (palliative or adjuvant chemotherapy); (2) tumors resistant to first-line local and systemic therapies (palliative chemotherapy); (3) patients with comorbidities that rule out first-line systemic and/or local treatments (palliative or adjuvant chemotherapy); (4) patients whose guardians decline first-line systemic and/or local treatments (palliative or adjuvant chemotherapy).

Despite the clinical benefits of MC, several important limitations warrant consideration. The delayed onset of therapeutic effects can reduce its suitability for rapidly progressing tumors, where prompt intervention is critical. This lag in response may limit its use as a sole therapy in aggressive cancer cases. Additionally, the lack of standardized protocols tailored to specific tumor types leads to variability in treatment regimens, hindering consistency in clinical indications and complicating comparative analysis across studies.

Owner compliance is another significant challenge, as the continuous daily oral administration of MC demands a high level of commitment and understanding from pet owners. Variability in adherence can negatively impact treatment efficacy and overall outcomes. This is compounded by the necessity for regular and often frequent hematological and biochemical monitoring to detect potential toxicities, especially with drugs such as cyclophosphamide and lomustine, which may impose additional burdens on both owners and veterinary teams.

These factors underscore the need for individualized treatment planning those accounts for tumor biology, patient condition, and owner capacity. Moreover, they highlight the importance of further controlled studies to establish optimized, evidence-based MC protocols that balance efficacy, safety, and practicality within veterinary oncology practice. Table 2 and Table 3 provide a summary of the main clinical studies evaluating the indications of MC in veterinary oncology, including tumor types, treatment protocols, and outcomes.

### Critical Analysis of the Studies and Levels of Evidence

To evaluate the methodological strength of the studies included in this review, we adapted the hierarchy of evidence proposed by the Center for Evidence-Based Medicine (CEBM) at the University of Oxford [47]. CEBM provides a structured approach for ranking clinical research based on study design, ranging from high-level randomized controlled trials to low-level expert opinion or case reports. It is widely used in evidence-based health sciences to assess the robustness and applicability of published data.

Given the heterogeneity of study designs in veterinary oncology, and the limited number of randomized controlled trials, we proposed a simplified, adapted version of the CEBM scale (Table 4). Because this article is not a systematic review or meta-analysis, but a narrative synthesis of heterogeneous studies in veterinary oncology, we employed this simplified adaptation of the CEBM scale. The proposed version uses a five-point ordinal score (1–5), based on several methodological parameters, including study design (e.g., prospective vs. retrospective), presence and adequacy of control groups, use of randomization and blinding, sample size, standardization of inclusion criteria and treatment protocols, and the clarity and objectivity of clinical outcome measurements. This approach provides a pragmatic and useful method to interpret the current level of evidence in the absence of standardized grading systems tailored for veterinary clinical trials.

When applying this adapted scoring system to the 20 studies identified in dogs, none were classified as level 1 or 2, highlighting the absence of randomized controlled trials in the veterinary literature on MC. A total of 8 studies (40%) were classified as level 3, comprising retrospective studies with control groups or single-arm prospective trials with methodologically acceptable but potentially biased designs. The majority of studies, 12 (60%), were classified as level 4, and no study was classified as level 5. Although most of level 4 studies included a control group, their methodological limitations precluded a higher score. These limitations included heterogeneous co-interventions (e.g., use of multiple chemotherapeutic agents or targeted therapies), lack of standardized outcome definitions, and incomplete or non-systematic follow-up data.

Regarding the feline studies, two (67%) were classified as level 4 and one as level 5 (33%). The only study classified as level 5 was a retrospective case series that included cats with a variety of tumor types, making interpretation of outcomes more difficult. The study exhibited several critical methodological limitations, such as the absence of a control group, a very small sample size, and substantial heterogeneity in both treatment protocols and tumor histology [46].

Besides the lack of prospective randomized studies, one of the most frequent limitations identified was the widespread use of combination protocols. Among the 23 studies evaluated, 19 (82.6%) combined MC with other agents, such as NSAIDs, thalidomide, or tyrosine kinase inhibitors (e.g., toceranib). In these cases, the individual contribution of MC to clinical outcomes cannot be clearly isolated from the effects of these concurrent therapies.

Moreover, meaningful comparisons between studies are further limited by substantial methodological heterogeneity. Differences in study design (retrospective vs. prospective), patient demographics, tumor types and stages, and the frequent use of multimodal protocols hinder the standardization of conclusions. The lack of uniform inclusion criteria, treatment regimens, and outcome definitions further complicates inter-study comparisons. These constraints should be explicitly acknowledged, especially for researchers intending to conduct meta-analyses or derive generalized clinical recommendations.

This analysis therefore reinforces the urgent need for prospective, controlled studies, ideally randomized, with well-defined inclusion criteria, standardized protocols, and robust clinical endpoints to more precisely validate the role of metronomic chemotherapy in the oncologic management of dogs and cats.

In the next section, a more in-depth review of the use of MC in different tumor types will be carried out.

## 5. Epithelial Neoplasms

In veterinary oncology, MC has emerged as a promising palliative and adjunctive strategy for treating epithelial tumors in dogs, which are often aggressive and difficult to manage with standard therapies. The following sections examine the use of MC across different epithelial tumor types, focusing on its efficacy, tolerability, and potential to improve survival and the quality of life.

### 5.1. Mammary Carcinomas

Several studies have investigated the use of MC in dogs with mammary carcinomas, particularly in advanced-stage or high-grade tumors. In one prospective study involving stage IV or grade II/III carcinomas, daily oral cyclophosphamide following MTDC with carboplatin significantly improved survival, with manageable hematological toxicities and no impact on the quality of life [22].

Another prospective trial evaluated 58 dogs with various histological subtypes of mammary neoplasms, comparing surgery alone, surgery followed by MTDC, and surgery combined with MC. The group receiving carboplatin followed by thalidomide achieved the longest MST (845 days), while MC with cyclophosphamide and firocoxib also improved outcomes compared to controls. Adverse effects were generally mild, though sterile hemorrhagic cystitis occurred in some dogs receiving cyclophosphamide, requiring temporary discontinuation and anti-inflammatory support [20].

A retrospective study [36] focused on inflammatory mammary carcinoma found that MC with cyclophosphamide, toceranib, and a COX-2 inhibitor significantly extended survival and delayed disease progression compared to anti-inflammatory therapy alone. Despite higher rates of mild to moderate gastrointestinal and hematological adverse effects, all dogs receiving MC demonstrated clinical benefit. These results highlight the potential of MC as a beneficial adjunct therapy in dogs with inflammatory mammary carcinoma, warranting further prospective investigations.

A retrospective study focused on inflammatory mammary carcinoma found that MC with cyclophosphamide, toceranib, and a COX-2 inhibitor significantly extended survival and delayed disease progression compared to anti-inflammatory therapy alone. Despite higher rates of mild to moderate gastrointestinal and hematological adverse effects, all dogs receiving MC demonstrated clinical benefit, including objective responses [36].

Overall, the available evidence supports the potential benefits of MC in managing malignant mammary tumors in dogs, particularly in advanced-stage or inflammatory cases. However, the heterogeneity in study designs, drug combinations, and treatment durations underscores the need for standardized protocols to optimize therapeutic outcomes and ensure patient safety.

The 2023 consensus on canine mammary tumors suggested cyclophosphamide and doxorubicin as possible adjunctive treatments for high-grade or aggressive tumors [48]. Nevertheless, it is important to clarify that doxorubicin is not used in MC due to its cumulative cardiotoxicity and pharmacokinetic profile. Future studies should aim to refine histology-specific indications for MC and establish evidence-based guidelines to improve its clinical indication.

### 5.2. Adenocarcinoma of the Apocrine Gland of the Anal Sac

Chemotherapy in dogs can be managed using MC protocols in cases of advanced disease or when conventional treatments are ineffective. In a study involving three dogs diagnosed with anal sac carcinoma and treated with chlorambucil at a dose of 4 mg/m^2^/day orally, two of the dogs achieved SD, while one experienced PD. The disease progression-free interval varied from 2 to 30 weeks, and the survival time (ST) ranged from 2 to 63 weeks [49]. These results suggested that the therapy indicates possible advantage of providing tumor control in certain cases, even in animals with advanced disease.

A separate study assessed various types of neoplasms in dogs, employing lomustine in a metronomic regimen, including one case of anal sac carcinoma. The administered dose was 2.84 mg/m^2^ orally, given daily, with ongoing hematological and biochemical monitoring. The therapy was well tolerated, exhibiting mild to moderate adverse effects—primarily involving the gastrointestinal system, thrombocytopenia, and elevated alanine aminotransferase levels—without any serious cases of liver disease [30]. These findings suggest that the MC with lomustine may offer a potential therapeutic benefit for dogs with anal sac carcinoma, particularly in cases of advanced or refractory disease, or when other treatment options are unavailable. However, further studies with larger sample sizes and controlled designs are necessary to confirm its safety and efficacy in this specific tumor type.

Mitchell et al. [49] evaluated MC for anal sac carcinoma in one dog, using toceranib at 2.75 mg/kg orally every other day alongside cyclophosphamide at 15 mg/m^2^/day. The protocol was well tolerated, with mild adverse effects primarily in the gastrointestinal and hematological systems. Toceranib significantly reduced regulatory T lymphocytes, and its combination with cyclophosphamide elevated serum interferon-γ levels, suggesting a potential restoration of the immune response [50].

### 5.3. Urothelial Carcinoma (UC)

Due to its location, UC is generally not amenable to surgical removal, and the efficacy and adverse effects of RT remain unclear. Chemotherapy, either alone or in combination with cyclooxygenase (COX) inhibitors, is the standard treatment for unresectable or metastatic cases [39].

A prospective clinical study [31] evaluated the efficacy and toxicity of metronomic oral chlorambucil administration in dogs with refractory UC or whose owners refused conventional treatments. Thirty-one dogs received chlorambucil at a dose of 4 mg/m^2^ orally every 24 h, resulting in the following responses: 3% PR, 67% SD, and 30% PD. The median time to progression was 119 days, while the MST was 221 days. Adverse effects were rare and generally mild, with only one case necessitating treatment interruption. The protocol indicates a possible advantage and the capacity to stabilize the disease in 70% of cases, rendering it a reasonable alternative for dogs with UC after conventional treatments have failed.

In one treatment case for UC in a female dog, surgical excision of the lesion was followed by MTDC with carboplatin at 250 mg/m^2^ intravenously every 21 days over six sessions. Following completion of MTDC and in the absence of recurrence, MC with cyclophosphamide at 15 mg/m^2^/day orally, for six months was implemented. This multimodal regimen resulted in prolonged tumor control, with disease-free survival of 21 months [51].

In a retrospective study involving 35 dogs with UC, MC was used as a rescue treatment in only one patient, who received chlorambucil at a dose of 4 mg/m^2^/day as a continuous oral dose. Due to the small sample size, a comprehensive evaluation of its efficacy was not possible. In this case, MC proved to be safe, with no significant adverse events; however, conventional MTDC protocols using gemcitabine or vinblastine, combined with COX inhibitors, demonstrated superior disease control [39].

In human studies, cyclophosphamide is not recommended for patients with transitional cell carcinoma or UC tumors due to its urocarcinogenic potential. Research has demonstrated that even a single dose can significantly increase the risk of tumor recurrence in the bladder and upper urinary tract. Furthermore, it is associated with the development of aggressive tumors, including renal pelvis carcinoma and invasive bladder carcinoma [38].

Furthermore, a systematic review [40] reported that about half the cases of bladder cancer associated with cyclophosphamide were invasive at diagnosis, carried high tumor-related mortality, and had an average interval of 10 years between the start of treatment and diagnosis. Thus, cyclophosphamide should be avoided in patients with a history or risk of UC, given the risks of tumor induction and long-term recurrence.

Although cyclophosphamide has been explored in some veterinary studies as part of MC protocols for UC, this approach raises significant safety concern. As highlighted by existing evidence from human oncology, cyclophosphamide is recognized for its urothelial carcinogenic potential and is contraindicated in patients with a history or risk of UC due to its association with tumor induction, recurrence, and aggressive behavior.

This presents a clinical contradiction when the same agent is considered for treating a tumor type it may potentiate. Given this risk, the use of cyclophosphamide in canine patients with UC should be avoided. Instead, safer alternatives such as chlorambucil should be emphasized and prioritized in MC protocols for these cases.

### 5.4. Liver Carcinomas

A prospective study by Marconato et al. [35] reported on dogs with unresectable hepatocarcinoma that received MC orally with thalidomide (2 mg/kg/day), piroxicam (0.3 mg/kg/day), and cyclophosphamide (10 mg/m^2^/day) (n = 6). The study found a mean time to progression of 27 days and a median overall survival of 32 days. These results indicated a low response to MC in treating carcinomas, specifically hepatocarcinomas.

The literature contains only one case report describing a primary hepatic carcinoma of neuroendocrine origin in a dog—an uncommon histological variant marked by diffuse hepatic changes that render complete surgical resection impossible. In such cases, the use of adjuvant chemotherapy, including MC, is recommended. In this report, we employed a protocol involving doxorubicin at a MTDC of 30 mg/m^2^, administered intravenously every 21 days for a total of five sessions, followed by MC with cyclophosphamide (13 mg/m^2^/day) administered orally. Additionally, toceranib phosphate was administered orally at a dose of 2.5 to 6 mg/kg on Mondays, Wednesdays, and Fridays. Signs of liver disease progression were observed after 10 months of treatment, ultimately leading to euthanasia 15.5 months (465 days) after therapy initiation [52].

Metronomic chemotherapy has shown limited efficacy in cases of canine hepatocarcinoma, with very short mean progression times and overall survival. However, in rare instances such as neuroendocrine liver carcinoma, an MC regimen combined with MTDC and toceranib can be considered a complementary approach to tumor control, notably when surgical resection is not feasible.

### 5.5. Primary Lung Carcinomas

Primary pulmonary neoplasia in dogs is an uncommon condition characterized by aggressive behavior and poor prognosis, particularly in advanced cases with metastases or when surgical resection is not possible [53].

Given the limitations of conventional therapies, such as surgery and MTDC, MC has been explored as an alternative approach. In a recent prospective study [33], 25 dogs were treated solely with MC, receiving cyclophosphamide 10 mg/m^2^/day or every other day, piroxicam 0.3 mg/kg/day, and thalidomide 2 mg/kg/day, administered orally. The clinical response was deemed satisfactory, with 16% of the dogs achieving PR and 76% maintaining SD, resulting in a clinical benefit rate of 92%. The median time to progression was 172 days, and the median survival time reached 139 days, significantly exceeding the outcomes obtained with surgery alone, MTDC, or no treatment.

The protocol suggested a potential benefit and was well tolerated, with mostly mild to moderate adverse effects, highlighting its potential as a safe and effective approach in the palliative management of advanced primary lung neoplasms [54]. These findings suggest that MC may be a potential strategy for prolonging survival and enhancing the quality of life in dogs with advanced primary lung carcinoma.

### 5.6. Metastatic Lung Carcinomas

Marchetti et al. [32] used MC as a first-line treatment for dogs with spontaneous metastatic tumors, including cases of lung adenocarcinoma. The protocol involved daily oral administration of cyclophosphamide 25 mg/m^2^ and celecoxib 2 mg/kg. This combination was chosen due to the antiangiogenic activity of cyclophosphamide at low doses and the anti-inflammatory and antitumor potential of celecoxib. Treatment continued until disease progression occurred.

Of the 15 dogs treated, 1 (7%) achieved CR and 5 (33%) had SD. The median overall survival was 3.39 months, a duration considered noteworthy given the advanced stage of the patients. Importantly, there was no significant hematological or metabolic toxicity, and the quality of life was reported to improve significantly after 30 days of treatment, with a reduction in clinical morbidity scores [32].

## 6. Mesenchymal Neoplasms

Sarcomas develop from the malignant transformation of mesenchymal cells and can arise in various anatomical sites. Soft tissue sarcomas (STS) are considered a heterogeneous group of tumors that originate from various tissues but share similar pathological characteristics and clinical behaviors. However, some sarcomas are excluded from this classification due to their more aggressive biological behavior and distinct histological characteristics (e.g., hemangiosarcoma, osteosarcoma, histiocytic sarcoma, and oral fibrosarcoma) [55].

### 6.1. Soft Tissue Sarcomas (STS)

Effective local control is crucial for the therapeutic success of STS. Surgery, either alone or in combination with RT or electrochemotherapy, is the most commonly recommended treatment [56,57]. However, certain negative prognostic factors can impede successful local treatment: (1) high-grade tumors; (2) locations challenging for complete resection; (3) recurrence following initial intervention; and (4) the presence of distant metastases [58]. In these cases, MC emerges as a therapeutic alternative in both palliative and adjuvant scenarios.

The adjuvant MC with cyclophosphamide (10 mg/m^2^ orally, every 24 h) and piroxicam (0.3 mg/kg/day orally) was retrospectively evaluated in dogs with incompletely resected STS (n = 30; 77% grade II), achieving a disease-free period superior to that obtained in the group treated with surgery alone (n = 55; 68% grade II) with a minimum of 410 days vs. 211 days, respectively [23]. However, the retrospective nature of the study and the lack of randomization should be considered when interpreting these findings.

The use of MC as an adjunct to hypofractionated RT (5 × 6 Gy) was retrospectively evaluated in 50 dogs with macroscopic STS that were unresectable or recurrent. The CT protocol consisted of cyclophosphamide (7 mg/m^2^ orally, every 48 h), thalidomide (1–2 mg/kg/day orally), and piroxicam (0.3 mg/kg/day orally). The results demonstrated significantly longer survival in patients who received CT after RT compared to those treated with RT alone (757 vs. 256 days, respectively). However, it is important to note that there was no difference in the progression-free period, and survival may be influenced by other factors unrelated to the disease [41].

Although new prospective and randomized studies are warranted, the results, to date, support the use of MC in dogs with STS in selected cases. It should be considered primarily in instances of advanced disease, including recurrent and/or metastatic tumors, as well as incompletely resected tumors.

### 6.2. Splenic Hemangiosarcoma

Given its high metastatic potential and aggressive nature, the treatment of splenic hemangiosarcoma (HSA) involves a combination of splenectomy with systemic adjuvant therapies to extend disease-free intervals and enhance survival rates [59].

Two retrospective studies evaluated the use of MC as an adjuvant to surgery (with cyclophosphamide or chlorambucil) in dogs with splenic HSA (stages I and II) and reported comparable or superior survival rates compared to those treated with adjuvant MTDC based on anthracyclines (doxorubicin, liposomal encapsulated doxorubicin, or epirubicin) [17,27]. It should be noted that other drugs, such as etoposide or thalidomide combined with piroxicam, have been associated with MC. Nevertheless, the sample sizes for groups treated with MC were small in both studies, necessitating further research to substantiate these findings. The MST and time to progression for this group were significantly lower than those of the group treated with adjuvant MTDC (134 vs. 52 days and 140 vs. 58 days, respectively), although there was a considerably lower degree of toxicity. These results suggest that the treatment of stage III splenic HSA should include adjuvant MTDC to achieve better disease control. However, maintaining the quality of life is a crucial consideration for patients with advanced disease [26].

Another retrospective study assessed MC with cyclophosphamide (10–15 mg/m^2^/day or EOD orally) as an adjuvant to surgery in a larger group of dogs with metastatic splenic HSA (stage III), alongside NSAIDs such as piroxicam or meloxicam (standard dose, every 24 h), with or without thalidomide (2–4 mg/kg/day orally). The MST and time to progression for this group were significantly lower than those of the group treated with adjuvant MTDC (134 vs. 52 days and 140 vs. 58 days, respectively), although there was a considerably lower degree of toxicity. These results suggest that the treatment of stage III splenic HSA should include adjuvant MTDC to achieve better disease control. However, maintaining the quality of life is a crucial consideration for patients with advanced disease [26].

The effectiveness of incorporating MC into adjuvant MTDC protocols has been examined in various studies, yet the results remain inconsistent. To date, findings indicate that MC with cyclophosphamide (10–15 mg/m^2^, orally, every 24 h, or 25 mg/m^2^ every 48 h), combined with or without etoposide and NSAIDs, in conjunction with the conventional adjuvant protocol (doxorubicin, 25–30 mg/m^2^, intravenously, every 21 days), either simultaneously or subsequently, resulted in a slight improvement or no significant improvement in survival for dogs with splenic HSA (stages I, II, and III) [21,42,43].

In conclusion, doxorubicin protocols remain the most effective adjuvant treatment for canine splenic HSA. In addition, MC may be considered as an alternative for selected cases, such as (1) patients with a lower tolerance to the side effects of MTDC; (2) patients with cardiac comorbidities; and (3) when owners prefer less intense treatment protocols.

### 6.3. Osteosarcoma (OSA)

Osteosarcoma (OSA) is characterized by its highly aggressive and metastatic biological behavior, with most affected dogs already harboring micrometastases at the time of presentation. In light of this, the concurrent use of MTDC as an adjuvant to amputation surgery is the first line of treatment; however, the prognosis for these patients remains guarded, with 90% of dogs succumbing to metastases [60].

Given that alterations in the tumor microenvironment play a significant role in metastatic progression, MC has emerged as a complementary therapeutic modality in the treatment of canine OSA through the modulation of immune responses and antiangiogenic effects. A retrospective study was conducted to compare dogs with OSA treated with surgery and MTDC (carboplatin, 300 mg/m^2^, IV, every 21 days), with and without the addition of MC using cyclophosphamide (15 mg/m^2^/day orally) combined with meloxicam (0.1 mg/kg/day orally) as a maintenance treatment. No significant difference in survival and progression-free time was observed between the groups [24].

## 7. Round Cell Tumors

The literature remains sparse regarding the indication of MC in veterinary medicine as a therapeutic alternative for neoplasms of round cell origin, such as mast cell tumors, transmissible venereal tumor, and plasmacytoma. 

In this context, there is a need for more robust and methodologically sound studies to validate the various therapeutic protocols involving MC. The establishment of standardized clinical guidelines may facilitate its wider, safer, and more systematic implementation in veterinary oncologic practice [3].

### 7.1. Mast Cell Tumors

A study assessed the efficacy and adverse effects of MC with lomustine compared to the conventional vinblastine and prednisone protocol in dogs with cutaneous mast cell tumor. The study included 32 dogs randomly assigned to two experimental groups. The first group was administered vinblastine (2 mg/m^2^) in combination with prednisone for 12 sessions, while the second group received lomustine (2.84 mg/m^2^, orally) in a metronomic regimen over four months. Most tumors (67.5%) were classified as grade II/low grade. Results indicated that the lomustine and prednisone protocol did not exhibit superiority in survival time or tumor recurrence-free interval when compared to the vinblastine and prednisone treatment [44].

### 7.2. Canine Transmissible Venereal Tumor (CTVT)

Currently, vincristine sulfate in MTDC is regarded as the therapy of choice, demonstrating high efficacy in controlling canine transmissible venereal tumor (CTVT) [61].

In a study [45] evaluating the hematological and biochemical effects of conventional and MC using vincristine sulfate for the treatment of canine transmissible venereal tumor, twelve dogs were selected and divided into two groups: G1, treated with vincristine (0.75 mg/m^2^, once a week), and G2, with a metronomic regimen (0.25 mg/m^2^, three times a week) until tumor remission. Conventional CT resulted in leukopenia due to neutropenia and normocytic normochromic anemia in 50% of the dogs. Metronomic treatment, however, preserved the leukogram and showed only a slight increase in BUN levels.

Metronomic treatment, however, preserved the leukogram and resulted in only a slight increase in BUN levels [45]. These findings suggest that MC may be possibly effective in oncologic treatment, while also exhibiting lower hematological toxicity, highlighting its potential as a promising therapeutic alternative.

### 7.3. Plasma Cell Tumors

Plasma cell tumors encompass a spectrum of diseases characterized by the clonal proliferation of plasma cells or homogeneous immunoglobulin-producing B cells [62]. In dogs, these tumors predominantly present as solitary cutaneous masses, generally without systemic clinical signs or laboratory abnormalities, and are often curable with complete surgical excision. However, extramedullary plasmacytomas may also affect visceral organs such as the gastrointestinal tract, lungs, spleen, kidneys, and in these locations, they may exhibit malignant behavior [63,64]. In contrast, in cats, cutaneous plasmacytomas are more commonly associated with bone marrow or other organ involvement, indicating potentially more aggressive biological behavior [65].

In the case report by Rezk et al. [66] a nine-year-old mixed-breed female dog was diagnosed with an oral plasmacytoma following marginal resection of a lingual mass and confirmation via immunohistochemistry. Due to the owner’s refusal of further surgical intervention to achieve clean margins and address a second lesion, a metronomic chemotherapy protocol using melphalan (1.5 mg/m^2^/day orally) was implemented for three months. After a two-year follow-up, no signs of disease progression were noted.

Although these findings are encouraging and suggest that MC with melphalan may offer therapeutic benefit in managing oral plasmacytomas when complete surgical excision is not possible, it is important to emphasize that this evidence stems from a single case report. Therefore, its applicability to broader clinical settings remains uncertain. Prospective studies with larger sample sizes and rigorous methodologies are required to validate the efficacy and safety of this approach.

## 8. Malignant Neoplasms of the Oral Cavity

Melanomas are the most common malignant neoplasms in the oral cavity of dogs. Surgery and RT are excellent options for local control; nonetheless, adjuvant immunotherapy or chemotherapy is indicated for patients at risk of tumor dissemination following incomplete excision due to the tumor’s high metastatic potential [67].

In a retrospective study [32], which evaluated the use of metronomic cyclophosphamide for treating dogs with various types of neoplasms, one dog had stage IV oral melanoma. This patient had not previously undergone any other therapeutic modalities and was treated with cyclophosphamide 25 mg/m^2^/day and celecoxib 2 mg/kg/day administered orally, which resulted in a 4-month disease-free survival. However, the absence of control groups and direct comparisons with other therapeutic modalities limits the evaluation of the real efficacy of MC. Also, oral lomustine at 2.84 mg/m^2^ has been used in a MC in two dogs with oral melanoma that had previously undergone palliative RT but showed a poor response [30].

In a study [68], out of 32 dogs with oral melanoma who received adjuvant chemotherapy after surgical excision, only 4 received MC, but the protocol of drugs and doses was unspecified. Consequently, the real benefit of MC in dogs with oral melanoma remained undetermined.

A retrospective study [2] included 12 dogs histologically diagnosed with malignant oral neoplasms, including fibrosarcoma, amelanotic melanoma, squamous cell carcinoma, adenocarcinoma, acanthomatous ameloblastoma, oral plasmacytoma, chondrosarcoma, neurofibrosarcoma, and oral melanoma. In these cases, owners declined other therapies considered as first-line, such as surgery or RT, and the metronomic protocol was evaluated only in a palliative context.

All dogs received cyclophosphamide at an average dose of 20.5 mg/m^2^ every 24 h (range 15–25 mg/m^2^/day), with 50% of cases also receiving NSAIDs. In 66.7% of cases, piroxicam (0.3 mg/kg/day) was selected for combination therapy. In 50% of the patients treated, a clinical benefit was observed after 30 days; this benefit was defined as a PR or SD. No dog achieved CR. The MST was 155 days (21–529 days) [2].

Regarding adverse effects, over 33% of dogs with oral neoplasms undergoing palliative MC with cyclophosphamide developed sterile hemorrhagic cystitis, occurring between 60 and 170 days after starting therapy. This was the most reported adverse event, followed by mild gastrointestinal effects such as vomiting and diarrhea. In all instances where adverse effects occurred, discontinuation of MC was sufficient to resolve the complications [2].

In conclusion, the real benefits of MC in the palliative context for malignant oral tumors have not yet been widely established, and dogs with oral melanomas or other malignant oral neoplasms should receive local treatment and adjuvant MTDC whenever possible. Furthermore, more prospective, randomized, placebo-controlled studies are needed to better elucidate the true benefit of MC in these neoplasms.

## 9. Gliomas

Gliomas are intracranial tumors affecting both dogs and cats. Generally, they are less common than meningiomas and occur more frequently in dogs than in cats, with any breed or size of dog being susceptible, although brachycephalic breeds are particularly affected [25].

Surgical treatment of gliomas poses challenges due to their location and the significant neoplastic infiltration into brain tissue, making complete removal difficult and necessitating complementary therapies. In human patients, experimental studies have concluded that MC with chlorambucil can produce a direct cytotoxic effect by increasing the drug concentration in tumor cells, identifying this drug as a potential target for tumor control [69].

A noteworthy clinical trial investigated the efficacy and safety of MC in combination with chlorambucil in canine patients with gliomas, as dogs serve as natural models for studying human gliomas [25]. In this study, eight canine patients with gliomas received oral MC with chlorambucil at a dose of 4 mg/m^2^ every 24 h during the preoperative period, combined with lomustine at 60 mg/m^2^ every four weeks postoperatively. Chlorambucil concentrations were measured in the surgically removed tumor and cerebrospinal fluid. The study indicated that some tumors might exhibit alterations in the blood–brain barrier, as the median chlorambucil concentration in brain tissue was about 37% of the serum concentration; however, direct cytostatic effects of chlorambucil, as observed in human experimental studies, were not evident [69,70].

Metronomic treatment in dog’s post-surgical tumor removal was well tolerated, with a low incidence of adverse effects. In the group treated with metronomic therapy based on chlorambucil and lomustine, the most common adverse effects were hematological and dose dependent. After chronic therapy, three out of eight dogs developed asymptomatic thrombocytopenia. In this study, the median disease-free progression was 253 days, and the MST was 257 days for the group treated with surgery and metronomic therapy consisting of chlorambucil and lomustine. These results exceeded the MST observed with palliative therapy, lomustine alone with surgery, or temozolomide alone or with surgery, although the longest MST was achieved with the combination of hypofractionated RT and lomustine. Additionally, an interesting finding from this study is that most dogs undergoing metronomic therapy with chlorambucil experienced a longer seizure-free interval compared to the pre-chemotherapy period [25].

According to some studies, adjuvant MC with chlorambucil and lomustine may be a complementary alternative to promote an improved quality of life, control of neurological signs, and increased survival in dogs diagnosed with gliomas [25,70]; however, more prospective, controlled, and randomized studies are needed, since the available studies are mostly retrospective, with low sample size, lacking histological confirmation and tumor classification and greater homogeneity of protocols.

## 10. Metronomic Chemotherapy in Cats

Studies on MC in felines are relatively recent, emerging approximately a decade after those conducted in dogs [1]. These studies remain limited, possibly due to challenges in continuous oral drug administration. However, the available evidence suggests potential efficacy in cats, indicating antiangiogenic and immunomodulatory effects alongside a lower rate of adverse events compared to MTDC, as observed in canine studies [1,28].

Metronomic chemotherapy protocols in cats share therapeutic objectives with those in dogs. These protocols can serve as adjuvant therapy within a multimodal approach to prolong remission or control microscopic disease [3]. Additionally, they may be employed as the primary treatment for advanced or disseminated diseases, providing palliative care to enhance the quality of life [28].

Furthermore, it could be an alternative as maintenance therapy after local tumor control, that is, being an option for feline patients whose owners refuse postoperative MTDC or in cases where the patient would not tolerate it, for example, due to the presence of serious comorbidities. With the attempt to delay the progression of the oncological disease, associated with lower rates of adverse effects [46].

Most published studies on MC in cats focus on its use in treating mammary neoplasms [28,71,72,73]. However, there are occasional case reports documenting its indication in abdominal HSA [46], urinary bladder HSA [74], squamous cell carcinoma [72,75], UC [76], STS [54], abdominal lymphangiosarcoma [72], and intraocular lymphoma [77], with variable outcomes and unfortunately there is still a low level of evidence for routine clinical indication.

A retrospective study on MC with cyclophosphamide in 24 cats diagnosed with diverse malignant neoplasms, including sarcomas, carcinomas, oral melanoma, and neuroendocrine tumors, revealed that most of the subjects received cyclophosphamide in combination with other drugs. The median dose was 14 mg/m^2^ per administration, with a weekly dose of 66 mg/m^2^. Most patients were treated with cyclophosphamide in combination with another drug, such as meloxicam, piroxicam, firocoxib, toceranib, or thalidomide. Only four patients received cyclophosphamide as monotherapy [46].

Cyclophosphamide was administered twice a week or more for a minimum period of 30 days. The main objective was to evaluate the efficacy of the treatment and the nature of potential adverse effects. To this end, patients were divided into two groups, one group having already received local or systemic therapy and the other limited group receiving only MC as palliative alternative [46]. A major limitation of this study is the lack of uniformity of the protocols in relation to drugs, doses, and frequencies, and the low number of patients who received only the metronomic protocol, which limits the attribution of positive and adverse effects to MC alone.

The study [46] reported hematological adverse effects in 8% of cases and renal effects in 4%. Long-term renal toxicity was noted in 20% of cases. Many cats were also treated with potentially nephrotoxic drugs, including NSAIDs and/or toceranib. Gastrointestinal adverse effects, including vomiting, diarrhea, and anorexia, were the most common and mostly self-limiting. Nonetheless, there was no systematic collection of data and standardized monitoring of adverse effects.

The results of the study [46] indicated that patients who received cyclophosphamide as an adjuvant MC, after receiving other types of associated local and/or systemic therapies, survived longer than the group of patients who received it as palliative therapy (progression-free survival = 297 days × 90 days [46]. However, as the study population was not homogeneous, with patients at different stages of the disease and undergoing different previous treatments, the interpretation of these results must be cautious.

A similar study [28] involving 23 cats treated with cyclophosphamide and meloxicam also reported mild to moderate gastrointestinal, hematological, and renal toxicities, which were generally self-limiting. However, in this retrospective study, the IRIS stage of renal disease was not determined prior to MC and the daily dose of meloxicam was higher than that usually recommended, which may have impacted renal adverse effects due to chronic use.

In another study [28] with felines with metastatic mammary carcinoma, different modalities of adjuvant treatment were compared, and three treatment groups were assigned: cats that received conventional chemotherapy, MC, and toceranib alone. The group that received MTDC had nine patients who received protocols involving doxorubicin 1 mg/kg every 3 weeks or carboplatin 250 mg/m^2^ every 3 weeks; the rate of occurrence of adverse effects was 66.7% in this group. The group that received toceranib phosphate (2.4–3.3 mg/kg on alternate days) involved 10 cats and the overall rate of adverse effects was 30%.

In the group of patients who received MC, the rate of adverse effects was only 20%. The group that received MC in this study consisted of 15 cats, of which 11 received cyclophosphamide (15 mg/m^2^/day orally) and 4 cats received chlorambucil (0.4–0.6 mg/kg on alternate days). However, there was no significant difference in survival time between the three groups [28].

According to the results found, it is concluded that the use of MC may demonstrate a certain therapeutic advantage in patients with disseminated mammary neoplasms, due to the lower incidence of adverse effects combined with the same survival time in relation to other therapeutic modalities, though it is important to emphasize the retrospective nature of the study and the small size of the groups.

A study [72] involving 11 cats with various neoplasms found that 73% had mammary carcinoma, while the remaining had squamous cell carcinoma (n = 1) and fibrosarcoma (n = 1). Six cats had metastatic disease and received MC with cyclophosphamide alone or in combination with toceranib or meloxicam in standard doses. The cyclophosphamide dose varied at 10–15 mg/m^2^, and gastrointestinal adverse effects, especially vomiting and anorexia, were most common. However, the retrospective nature, the lack of homogeneity between neoplastic types and phases of the disease, and the small sample size also stand out as potential limitations of the study, making it difficult to establish cause and effect relationships and, especially, to evaluate the real effectiveness of the protocols.

Notably, in studies involving cyclophosphamide, no cats developed hemorrhagic cystitis, a common side effect in dogs [46,78]. In dogs, the prevalence of hemorrhagic cystitis following cyclophosphamide administration is between 3.8 and 15% and may be reduced when administered with furosemide. However, in cats, the occurrence of this adverse effect is considered rare [27].

Similar to canines, MC has been investigated in feline patients with metastatic mammary neoplasms [79]. In this study, 73 cats with metastatic mammary carcinomas were evaluated, dividing them into 4 groups according to the type of treatment: MTDC (n = 9), MC (n = 15), palliative therapy (n = 10), and cats that did not undergo any treatment (n = 39). The main aim was to compare the group undergoing therapy with MTDC vs. MC. In this study, adjuvant treatments did not correlate with greater survival or disease-free time. However, it is important to consider a possible selection bias, since decisions about which therapy to choose for each case involved issues such as cost or personal preferences of the owners, providing non-homogeneous treatment groups regarding age and tumor stage.

Cats treated with MTDC were subjected to protocols involving administration of doxorubicin (1 mg/kg, intravenously, every 21 days) or carboplatin (250 mg/m^2^, intravenously, every 21 days). Cats undergoing palliative therapy received toceranib phosphate (2.4–3.3 mg/kg, every other day) while cats undergoing MC received cyclophosphamide (15 mg/m^2^, every day, orally) or chlorambucil (0.4–0.6 mg/kg, every other day, orally) [79].

Regarding the toxicity of these protocols, cats that received doxorubicin after surgery developed gastrointestinal adverse effects in 52.9%, while adverse effects were present in 39.1% of cats that received the metronomic protocol. There was no statistically significant difference regarding survival time between the treated groups, although cats that received MC had a longer survival time with a median of 75 days, compared with 58 days in the MTDC group, 63 days in palliative therapy, and 44 days in the untreated group [79].

However, cats undergoing MC had fewer adverse effects compared to the other treatment groups [2], but an in-depth comparison between the MC with cyclophosphamide and chlorambucil was unfortunately not available and would not have statistical power due to the low and different number of patients in each group.

In another study [80], a 12-year-old cat with a large, aggressive visceral HSA was treated with palliative MC monotherapy using cyclophosphamide (16.6 mg/m^2^/day). The cat survived for 10 months, exhibiting mild gastrointestinal signs and thrombocytopenia. However, it is important to highlight the lack of robust evidence regarding the use of MC in cats with visceral HSA, since this is only a case report.

Similarly, another investigation reported the use of cyclophosphamide (12.5 mg/m^2^/day) as adjuvant therapy for a 4.5-year-old cat with intraocular lymphoma or which the owners refused definitive therapy with enucleation. The patient underwent MTDC for induction treatment with vincristine, L-asparaginase and prednisolone, and for maintenance of treatment, MC was well tolerated. After one year from the beginning of the therapy the patient remained clinically well, with no signs of recurrence [77]. Although the result is interesting, it is worth noting that this is an isolated case report. There are no studies comparing the survival of patients treated with an induction protocol with MTDC followed by MC vs. induction with MTDC and only monitoring.

A third study involved a 4-year-old cat with vesicular hemangiosarcoma that was treated with adjuvant MC (cyclophosphamide 15 mg/m^2^/day, meloxicam 0.05 mg/kg/day, and thalidomide 1.7 mg/kg/day) after partial cystectomy. The cat remained recurrence-free and maintained a good quality of life after 896 days of monitoring [74].

The use of MC has also been evaluated for epithelial neoplasms such as squamous cell carcinoma. A case report [54] described a 7-year-old feline with a chronic ulcerated lesion on the nasal plane, diagnosed by biopsy with squamous cell carcinoma. Following surgical treatment, MC was successfully controlled with cyclophosphamide (10 mg/m^2^/day) and meloxicam (0.05 mg/kg/day) administered over five months, thereby enhancing quality of life.

In another study [81], a squamous cell carcinoma was detected extending from the nasal plane to the upper lip and the medial eyelid corners in a 12-year-old cat that was resistant to previous therapies, including cryosurgery and electrochemotherapy. Reconstructive surgery and nosectomy were performed, followed by MC with cyclophosphamide (12.5 mg/m^2^) and piroxicam (0.3 mg/kg). The patient was alive and free from relapse one year after initiating MC. However, further studies are required to validate the efficacy of MC as an adjuvant for squamous cell carcinomas, as outcomes may vary based on lesion characteristics, local therapy efficacy, and clinical staging.

In the study developed by Castro-López et al. [54], chlorambucil (2 mg/m^2^, three times a week) was associated with toceranib phosphate (1.67 mg/kg, 3 times a week) and meloxicam (0.01 mg/kg/day) in a 10-year-old feline diagnosed with abdominal lymphangiosarcoma. The patient presented PR, followed by SD for 54 days, with tumor recurrence occurring approximately 60 days after starting the protocol.

Although with the current data, we may infer that MC in cats may be related to the lower rates of adverse effects and lower therapy costs in relation to MTDC and may be a therapeutic possibility to prevent the progression of oncological disease, especially in cases of disseminated mammary neoplasms.

However, it is important to note that since most trials using MC in feline species are reports of isolated cases, or studies involving a small number of patients, we hope that more randomized, placebo-controlled studies will be developed, with homogeneous treatment groups evaluating the efficacy of the modality as adjuvant and palliative therapy in the various neoplasms that affect the species.

Current data suggest that feline MC is associated with lower adverse effects and reduced therapy costs relative to MTDC. It may also serve as an effective therapeutic option for preventing the progression of oncological diseases, particularly in cases of disseminated mammary neoplasms.

## 11. Biomarkers in Metronomic Chemotherapy

One of the greatest challenges in monitoring the response to MC is the lack of reliable biomarkers to identify patients most likely to respond, as well as to inform the formulation of individualized therapeutic protocols [10].

The urokinase-type plasminogen activator (uPA) system and its receptor (uPAR) have been investigated as potential therapeutic targets in both human and canine oncological diseases due to their role in angiogenesis, metastasis, and tumor invasion [81].

The activation of uPA with its receptor is directly associated with processes of cell survival, facilitating proliferation, migration, invasion, and tissue remodeling [81]. Furthermore, it is involved in complex mechanisms that may enhance metastatic potential [82].

A study evaluating uPAR expression in serum and tissue samples from dogs with osteosarcoma reported high uPAR expression. Although the prognostic value of this pathway was not as well-defined as in human studies [83,84], dogs with osteosarcoma exhibiting high uPAR levels showed lower progression-free survival and overall survival rates [82].

Currently, the literature on the role of the uPA/uPAR system as a biomarker in dogs receiving MC is limited. In one study, dogs with STS were assessed following treatment with metronomic protocols after surgical excision [83].

A dog exhibiting tumor volume reduction showed decreased serum and tissue levels of uPA/uPAR after initiating a metronomic protocol with cyclophosphamide. Nonetheless, generally, uPA/uPAR levels varied in the studied population, and in vitro expression of uPA/uPAR in cell lines did not change significantly following cyclophosphamide treatment [83].

We conclude that the actual impact of the uPA/uPAR pathway as a therapeutic biomarker in dogs receiving MC remains unknown. However, given its high expression in malignant tumors, it holds promise as potential.

## 12. Future Perspectives

Metronomic chemotherapy is a growing therapeutic area in veterinary oncology. As research advances, the expectation is for its usage to become more prevalent and substantiated, particularly within integrated and personalized protocols, prioritizing quality of life, reduced toxicity, and as an alternative therapeutic option for cancer patients [1].

There is an evident need for more robust and methodologically well-designed studies to validate various therapeutic protocols involving MC. The development of standardized clinical guidelines could facilitate its wider, safer, and more systematic indication in cancer treatment [3].

## 13. Conclusions

Metronomic chemotherapy emerges as a promising therapeutic approach in veterinary oncology, characterized by its low toxicity profile and multiple antitumor mechanisms. Its indication in dogs and cats has demonstrated encouraging clinical results, particularly in the treatment of neoplasms such as carcinomas, sarcomas, and hemangiosarcomas. This makes it a viable alternative for cancer patients who cannot tolerate conventional cytotoxic protocols.

Despite these advances, further clinical studies are necessary to consolidate MC in clinical practice by validating its efficacy across different tumor types and developing standardized protocols. However, its clinical integration must consider certain limitations, including the lack of protocol standardization, delayed onset of action, and challenges in owner compliance.

Additionally, inter-study comparisons remain limited due to methodological heterogeneity across available studies. Integration with adjuvant therapies and the identification of predictive biomarkers could enhance its effectiveness, promoting a more individualized and safer therapeutic approach in veterinary medicine.

## Figures and Tables

**Table 1:** Key pharmacological characteristics of common metronomic chemotherapy (MC) agents in veterinary oncology.

Drug	Dose (Orally)	Half-Life	Key Toxicities
Cyclophosphamide	10–25 mg/m^2^/day or 25 mg/m^2^/EOD	4–12 h (IV)	Myelosuppression (nadir ~7 d), sterile hemorrhagic cystitis, GI signs, alopecia
Chlorambucil	4 mg/m^2^/day	1.5 h (parent); 2.4 h (metabolite)	Cumulative myelosuppression
Thalidomide	5–10 mg/kg/day	4.8–17 h	Infrequent; caution due to unknown excretion route
Piroxicam	0.3 mg/kg/day	~12 h	GI toxicity, nephrotoxicity (especially in cats)
Doxycycline	5–10 mg/kg/twice a day	Variable	GI signs (especially in cats), staining of unerupted teeth in young animals

EOD: every other day; IV: intravenous; GI: gastrointestinal; h: hours; d: days.

**Table 2:** Clinical studies evaluating metronomic chemotherapy (MC) in dogs, with adapted CEBM evidence score *.

Tumoral Type	Nature of the Study (Number of Animals)	Treatment Group	Disease Setting	Metronomic Drug	MC-Associated Drugs	MTDC +/− Other Drugs	Response Evaluation and Survival Data	Adapted CEBM Score * (1–5)
Soft tissue sarcoma	Retrospective (n = 85) [23]	Sx + MC: n = 30; Sx alone: n = 55.	Microscopic	CYC (10 mg/m^2^/day orally)	Piroxicam	None	MC group achieved significantly longer DFI than control group (median not reached vs. 211 days, respectively).	3
	Retrospective (n = 50) [41]	RT + MC: n = 20; RT alone: n = 30	Macroscopic (palliative)	CYC (7 mg/m^2^/EOD orally)	Thalidomide and piroxicam	None	OST was significantly longer in RT + MC group when compared to RT alone group (757 vs. 286 days).	4
Splenic HSA	Retrospective (n = 33) [17]	Sx + MC: n = 9; Sx + DOX: n = 24.	Microscopic (stages I and II)	CYC (12.5–25 mg/m^2^/day orally)	Etoposide and piroxicam	DOX	MST was significantly longer in Sx + MC group than Sx + DOX group (178 vs. 133 days, respectively)	4
	Retrospective (n = 93) [27]	Sx + MC: n = 20; Sx + MTDC + MC (sequentially) n = 23; Sx + MTDC: n = 50.	Microscopic (stages I and II)	CYC (10–15 mg/m^2^/day or EOD orally) or chlorambucil (4 mg/m^2^/day orally)	NSAIDs (n = 23: Piroxicam, meloxicam or firocoxibe) and/or thalidomide (n = 3)	DOX, EPI or PL-DOX	MST and TTP did not differ between the groups (*p* > 0.05)	4
	Retrospective (n = 103) [26]	Sx + MC: n = 38; Sx + MTDC: n = 23; Sx alone: n = 42	Microscopic (stage III)	CYC (10–15 mg/m^2^/day or EOD orally)	NSAIDs (n = 38: Piroxicam or meloxicam) and/or thalidomide (n = 35)	DOX +/− CYC or Dacarbazine or EPI alone	MST and TTP were significantly lower in MC group compared to MTDC group (134 vs. 52 days e 140 vs. 58 days, respectively)	4
	Retrospective (n = 208) [42]	Sx + MC: n = 13; Sx + MTDC: n = 28; Sx + MTDC + MC (concurrently): n = 9; Sx + MTDC + MC (sequentially): n = 4; Sx alone: n = 154	Microscopic (89% stages II or III)	CYC (9.3–16 mg/m^2^/day orally)	NSAIDs (n= 12: Piroxicam, meloxicam or deracoxib)	DOX +/− CYC, cisplatin, ifosfamide or vincristine	Combination of MC and MTDC apperead to be more effective than these drugs alone, but improvement in ST was modest.	4
	Retrospective (n = 33) [43]	Sx + MTDC + MC (sequentially): n = 14; Sx + MTDC + MC (concurrent): n = 4; Sx + MTDC: n = 15	Microscopic (84% stages II or III)	CYC (10–25 mg/m^2^/day or EOD orally)	NSAIDs (n = 13): meloxicam (n = 11), piroxicam (n = 1) and deracoxib (n = 1)	DOX	PFS and OST were not significantly improved with MC addition.	4
	Retrospective (n = 61) [21]	Sx + MTDC + MC (concurrent): n = 22; Sx + MTDC: n = 39	Microscopic (68% stage II)	CYC (12–25 mg/m^2^/day or EOD orally)	NSAIDs (n = 13) or doxycycline (n = 5)	DOX +/− CYC	Addition of MC did not improve the outcome.	4
Osteosarcoma	Retrospective (n = 39). [24]	Sx + MTDC + MC (sequentially): n = 19; Sx + MTDC: n = 20	Microscopic	CYC (15 mg/m^2^/day orally)	Meloxicam (n = 18)	Carboplatin	OST and PFT did not improve significantly with MC addition.	4
	Retrospective (n = 86) [34]	RT + MC: n = 43; RT +/− MTDC: n = 43	Macroscopic.	Lomustine (2.84 mg/m^2^/day orally)	NSAID (n = 40)	Carboplatin, cisplatin, toceranib or masitinib	MST was not significantly improved with MC addition.	4
Mammary carcinoma	Prospective (n = 58) [20]	Sx + MTDC + MC (concurrently): n = 9; Sx + MTDC + thalidomide: n = 23; Sx + MTDC: n = 15; Sx alone: n = 11	Microscopic (59.2% stage IV)	CYC (15 mg/m^2^/day orally)	Firocoxib	Carboplatin and thalidomide	MST did not significantly differ between the groups.	3
	Prospective (n = 16) [22]	Sx + MTDC: n = 8; Grupo Sx + MTDC + MC (concurrently): n = 8	Microscopic (87.5% Stage III or IV and grade II)	CYC (12.5 mg/m^2^/day orally)	None	Carboplatin	MC addition did not improve significantly the MST.	3
Mammary inflammatory carcinoma	Retrospective (n = 16) [36]	MC + Toceranib + NSAIDs +/− Sx: n = 8; NSAIDs +/− Sx: n = 8	Microscopic (n = 10); Macroscopic (n = 6)	CYC (12.5 mg/m^2^/day orally)	NSAIDs (firocoxib, cimicoxib, meloxicam or piroxicam) and toceranib	None	Clinical benefit in all dogs of the multi-drug therapy, as well as significantly longer OST and TTP, compared to NSAIDs group.	4
Urothelial carcinoma	Prospective (n = 31). [31]	MC as sole therapy	Macroscopic (palliative: first-line or rescue therapy)	Chlorambucil (4 mg/m^2^/day orally)	NSAIDs (n = 25): piroxicam or firocoxib	None	PR (3%; n = 1); SD (67%; n = 20) and PD (30%; n = 9). Median PFT of 119 days and MST of 221 days.	3
Hepatocellular carcinoma	Prospective (n = 13). [35]	MC alone: n = 6; Sorafenib alone: n = 7	Macroscopic (palliative: first-line therapy; stages III-IV)	CYC (10 mg/m^2^/day orally)	Piroxicam and thalidomide	Sorafenib	MC group: SD (n = 3) and PD (n = 3); MST and TTP were significantly longer in sorafenib group.	3
Primary pulmonary carcinoma	Retrospective (n = 91) [33]	MC alone: n = 25; Sx alone: n = 36; MTDC alone: n = 11. No treatment: n = 19	Macroscopic (palliative: first-line therapy)	CYC (10 mg/m^2^/day or EOD orally)	Piroxicam and thalidomide	Vinorelbine, Carboplatin or Gemcitabine	MC group: PR (n = 4; 16%), SD (n = 19; 76%), and PD (n = 2; 8%). Clinical benefit of 92%. MST was similar between the groups.	4
Mast cell tumors	Prospective (n = 32) [44]	Sx + MC: n = 16; Sx + MTDC: n = 16	Microscopic (67.5% grade II/low grade)	Lomustine (2.84 mg/m^2^/day orally)	None	Vinblastine and Prednisone	No significant difference between ST and DFI between the groups.	3
Transmissible venereal tumors	Prospective (n = 12). [45]	MC alone: n = 6; MTDC alone: n = 6	Macroscopic	Vincristine (0.25 mg/m^2^/3x a week IV)	None.	Vincristine	MC provided fewer AEs and similar results compared to MTD vincristine.	3
Malignant oral tumors	Retrospective (n = 12) [2]	Non-applicable	Macroscopic disease (palliative: first-line therapy).	CYC (15–25 mg/m^2^/day orally)	Piroxicam, meloxicam or carprofen	None	DP in 83.3% and MST of 155 days. Clinical benefit of 50% (1st month) and 33% (2nd month).	4
Glioma	Prospective (n = 8) [25]	Non-applicable	Macroscopic disease (pre-Sx) and microscopic disease (post-Sx)	Chlorambucil (4 mg/m^2^/day orally)	Lomustine and prednisone	None	Median PFS and ST were 253 and 257 days, respectively.	3

HSA: hemangiosarcoma; MTDC: maximum tolerated dose chemotherapy; DOX: doxorubicin; CYC: cyclophosphamide; EPI: Epirubicin; PL-DOX: pegylated liposome encapsulated doxorubicin; MST: Median survival time; ST: Survival time; TTP: Time to progression; PFS: Progression-free survival time; OST: Overall survival time; PFT: Progression-free time; DFT: Disease-free time; RT: Radiotherapy; EOD: Every other day; NSAIDs: Non-steroidal anti-inflammatory drugs; SD: Stable disease; PD: Progressive disease; PR: Partial response; GI: Gastrointestinal; AE: Adverse events. * CEBM: Center for Evidence-Based Medicine. Evidence scores were adapted from the original CEBM levels to rank methodological strength (1 = highest; 5 = lowest).

**Table 3:** Clinical studies evaluating metronomic chemotherapy (MC) in cats, with adapted CEBM evidence score *.

Tumoral Type	Nature of the Study (Number of Animals)	Treatment Group	Disease Setting	Metronomic Drug	MC-Associated Drugs	MTDC +/− Other Drugs	Response Evaluation and Survival Data	Adapted CEBM Score (1–5)
Various tumor types **	Retrospective (n = 24) [46]	NA	Macroscopic (n = 17) and microscopic (n = 7)	CYC: 6–27 mg/m^2^ (median 14 mg/m^2^/day or EOD orally)	NSAIDs (n = 11): meloxicam, piroxicam or firocoxib +/− toceranib (n = 4) +/− thalidomide (n = 6)	None	Median PFS was 297 and 90 days for adjuvant and palliative groups, respectively	5
Mammary carcinoma	Retrospective (n = 137) [28]	Sx + MC + meloxicam: n = 23; Sx + MTDC: n = 34; Sx alone: n = 80	Microscopic (64% stage III)	CYC (15 mg/m^2^/day orally)	Meloxicam	DOX	Adjuvant MC did not improve survival. Differences in median DFI and OS were not significant	4
	Retrospective (n = 73) [28]	MC +/− Sx: n = 15; MTDC +/− Sx: n = 9; Toceranib +/− Sx: n = 10; Sx alone: n = 39.	Macroscopic metastatic disease (stage IV)	CYC (n = 11): 15 mg/m^2^/day orally or chlorambucil (n = 4): 0.4–0.6 mg/m^2^, EOD orally	None	DOX (n = 7) or carboplatin (n = 2) or Toceranib	Median TSS was 58, 75 and 63 days for groups, MTDC, MC and toceranib, respectively, but with no significant difference between them	4

TSS: Tumor-specific survival; MTDC: Maximum tolerated dose chemotherapy; CYC: cyclophosphamide; MST: median survival time; DFI: disease-free interval; PFS: progression-free survival time; OS: overall survival; EOD: every other day; NSAIDs: non-steroidal anti-inflammatory drugs; SD: stable disease; PD: progressive disease; PR: partial response. * CEBM: Center for Evidence-Based Medicine. Evidence scores were adapted from the original CEBM levels to rank methodological strength (1 = highest; 5 = lowest). ** Sarcomas (n = 13), carcinomas (n = 12), melanoma (n = 1), and neuroendocrine tumor (n = 1).

**Table 4:** Adapted classification of levels of evidence (based on CEBM *).

Adapted CEBM Score	Description	Key Characteristics
1	Very high evidence	Well-designed randomized controlled trial (RCT) with proper allocation and low bias risk.
2	High evidence	Prospective study with a control group, but without randomization or with some limitations.
3	Moderate evidence	Retrospective study with a control group, or single-arm prospective study with clear design.
4	Low evidence	Retrospective study without a control group, case series, or incomplete cohort data (e.g., major confounding factors, heterogeneous treatments, unstandardized follow up).
5	Very low evidence	Case report, expert opinion, or study with critical methodological flaws.

* CEBM: Center for Evidence-Based Medicine. This adaptation was developed to better assess non-randomized clinical studies in veterinary medicine, acknowledging the scarcity of robust randomized trials in the field.

## Data Availability

No new data were created.

## Abbreviations

COX-2	Cyclooxygenase-2
TNF-α	Tumor necrosis factor alpha
IL-6	Interleukin-6
